# New Appliances and Things Medical

**Published:** 1908-05-30

**Authors:** 


### NEW APPLIANCES AND THINGS MEDICAL.

[We shall be glad to receive at our Office, 28 & 29 Southampton Street, Strand, London, W.C., from the manufacturers, specimens of all new preparations and appliances which may be brought out from time to time.]

### ALEXINE.

(J. Chatelain, Usine and Depot, 15 Rue de Paris, Puteaux, Seine.)

The preparation before us is a granular one, similar pharmaceutically to many other French preparations. We draw attention to this because the method of preparing drugs in this form is an exceedingly useful one, the product being most palatable. The composition of Alexine is, however, unique in that according to the literature before us it consists of free phosphoric acid in a solid form. The importance of phosphoric medication in modern therapeutics is now recognised beyond dispute, and the vast number of new preparations of phosphorus and its derivatives recently put on the market is in itself ample evidence of the extensive use of this class of remedy. Alexine, however, possesses the advantage of containing phosphoric acid not in the form of phosphate but in the form of the acid itself. This property renders it likely to be especially useful, as it not only adds, by virtue of its phosphoric-acid content, to the phosphorus available to the body, but enables the body to utilise to better advantage the phosphorus of the food, and also to excrete more easily the useless inorganic phosphates. It in no sense acts as an alkali, but as an acid. We think it likely to be most useful in neurasthenia and conditions of malnutrition, and well worthy of an extensive trial at the hands of the profession.

### A NEW POWDER CARRIER.

An ingenious little receptacle for powder has lately been sent to us by the inventors. It was intended originally for the convenience of those ladies who like to carry about with them a supply of face-powder, and for this purpose it is no doubt suited, since it is small and cleanly. But its claim to our attention rests upon the statement that it has already been received with favour by dental surgeons and medical men, whose work entails the repeated washing of their hands at short intervals, and who thus have need of a clean and handy supply of drying powder. The "Poudro" is a nickel-plated tube about the size and shape of a small pencil-case. Any kind of powder can be poured into it, and when required a small quantity can be discharged by pressing the pointed end downwards against the hand or elsewhere. This forces back a small cap, previously held forward by a concealed spring, and allows a little powder to escape. The release of the spring, when the pressure is withdrawn, closes the tube again, and contamination and waste of the contents are thus prevented. The tube can be refilled without difficulty, and the mechanism is too simple to get out of order. Toilet powder is supplied; but medical men might find the "Poudro" handy for carrying a small supply of iodoform or other medicinal powder. The manufacturers are Thomas Christy and Company, 4, 10, and 12 Old Swan Lane, Upper Thames Street E.C.

### NEW INSTRUMENTS AND APPLIANCES IN GERMANY.

One of the most interesting features of the German Surgical Congress recently concluded at Berlin was the excellent exhibition of instruments and appliances, and of *x*-ray apparatus. Among the instruments, the visitor was at once struck by the large number of new types and modifications of old instruments, especially for intra-thoracic work and abdominal operations. Only a few notes are possible here, and if any one type deserves particular mention it is the large copper retractor invented by Trendelenburg. This is nickel-plated, and can be readily bent into any shape, concave or convex, and should prove of great service to the practitioner who cannot take with him half a dozen retractors bent at various angles. Another equally serviceable new modification is the "permanent screw" pin for fractures of the femur; a third is the light, one-toothed "towel forceps;" while yet another is the practical "general practitioner's pocket-case," made to fit into the waistcoat pocket, yet containing an armamentarium which one has hitherto associated with a large "dressing bag." The great cheapness of all the exhibits was surprising to an English practitioner accustomed to the high prices charged by instrument makers in this country. Thus a sterilisable box containing six hand-forged solid scalpels was priced at half a guinea. The *x*-ray apparatus was interesting because it represented the newest types of instruments, by means of which it is possible to take a photograph in the astonishingly short space of a quarter of a second. This modification is not unknown here, and is in use, we believe, in a few institutions. In Germany, however, the Grisonator method has been introduced in every *x*-ray room, and has practically supplanted the older apparatus. The photographs given by this instantaneous method are wonderfully sharp, clear, and distinct, and the apparatus only requires to be more widely known to win general recognition and appreciation.

